# Supplementary material for: Probiotics for the prevention of antibiotic-associated adverse events in children—A scoping review to inform development of a core outcome set
Source: PLoS One. 2020 May 29;15(5):e0228824. doi: 10.1371/journal.pone.0228824 (PMC7259577; doi:10.1371/journal.pone.0228824)
Supplement: S1 Fig — (PDF) [file pone.0228824.s001.pdf]

Supplementary Figure 1. Risk of bias summary for the included studies.

	Random sequence generation (selection bias)	Allocation concealment (selection bias)	Blinding (performance bias and detection bias)	Incomplete outcome data (attrition bias)	Selective reporting (reporting bias)	Other bias
Ahmad 2013	?	?	+	+	+	?
Akcam 2015	−	−	−	+	?	?
Arvola 1999*	+	?	+	−	?	+
Basnet 2017	−	−	−	+	?	?
Bin 2015	?	?	−	+	?	?
Correa 2005*	?	?	+	?	?	?
Dharani 2017*	?	?	?	+	?	?
Erdeve 2004*	+	?	?	−	?	?
Esposito 2018*	?	?	?	+	?	?
Fox 2015*	+	+	+	+	+	+
Georgieva 2015*	+	+	+	?	+	−
Hurduc 2009	+	+	−	+	?	?
Jindal 2017*	?	?	−	+	?	+
Jirapinyo 2002*	?	?	?	?	?	?
Korpela 2016	+	?	+	?	?	+
Kotowska 2005*	+	+	+	+	?	?
Kołodziej 2018*	+	+	+	+	+	+
Lionetti 2006	+	+	+	+	?	+
Merenstein 2009*	?	?	+	+	+	+
Okazaki 2016	+	+	?	?	?	?
Olek 2017*	+	+	+	+	+	−
Plewińska 2006	−	?	?	+	?	+
Ranasinghe 2008	?	−	−	+	?	?
Ruszczyński 2008*	+	+	+	+	?	+
Seki 2003	−	−	−	+	?	?
Shahraki 2017	+	−	−	+	+	?
Shan 2013*	+	+	−	−	?	?
Sykora 2005*	+	+	+	+	?	+
Szajewska 2009*	+	+	+	−	?	+
Szymański 2008*	+	+	+	+	?	?
Tankanow 1990*	?	?	−	−	?	−
Tolone 2012	?	−	−	+	?	?
Vanderhoof 1999*	+	?	+	+	?	?
Wang 2014	+	−	−	+	?	?
Zakordonets 2016*	+	?	−	+	?	−
Zoppi 2001	?	−	−	?	?	+

*studies with the risk of bias assessment derived from the recent Cochrane review
