# Supplementary material for: Probiotics for the prevention of antibiotic-associated adverse events in children—A scoping review to inform development of a core outcome set
Source: PLoS One. 2020 May 29;15(5):e0228824. doi: 10.1371/journal.pone.0228824 (PMC7259577; doi:10.1371/journal.pone.0228824)
Supplement: S1 Table — (DOCX) [file pone.0228824.s002.docx]

**S1 Table.** MEDLINE Search Strategy (Ovid MEDLINE(R) and Epub Ahead of Print, In-Process & Other Non-Indexed Citations, Daily and Versions(R))

| 1 | exp probiotics/ or probiotic*.mp. |
| --- | --- |
| 2 | exp lactobacillus/ or (lactobacill* or "l acidophilus" or "l casei").mp. |
| 3 | exp bifidobacterium/ or (bifidobacter* or "b infantis" or "b bifidum" or "b longum").mp. |
| 4 | exp saccharomyces/ or (saccaromyce* or "s boulardii").mp. |
| 5 | clostridium butyricum/ or clostridium difficile/ or (clostridium butyricum or clostridium difficile).mp. |
| 6 | streptococcus thermophilus/ or streptococcus thermophilus.mp. |
| 7 | enterococcus faecium/ or enterococcus faecium.mp. |
| 8 | or/1-7 |
| 9 | exp anti-bacterial agents/ |
| 10 | exp beta-lactams/ or exp macrolides/ or exp fluoroquinolones/ or exp tetracyclines/ or exp lincosamides/ or exp aminoglycosides/ or exp trimethoprim/ |
| 11 | (antibiotic* or anti biotic* or antimicrobial* or anti microbial* or antimycobial* or anti mycobial* or antimycobacteri* or anti mycobacteri* or antibacteri* or anti bacteri* or bacteriocid* or antiinfective* or anti infective*).mp. |
| 12 | (penicillin* or flucloxacillin* or amoxicillin* or clavula* or macrolide* or fluoroquinolone* or tetracycline* or lincosamid* or aminoglycosid* or trimethoprim*).mp. |
| 13 | or/9-12 |
| 14 | pediatrics/ |
| 15 | (infan* or newborn* or new-born* or perinat* or neonat* or baby or baby* or babies or toddler* or minors or minors* or boy or boys or boyfriend or boyhood or girl* or kid or kids or child or child* or children* or schoolchild* or schoolchild or adolescen* or juvenil* or youth* or teen* or underage* or pubescen* or pediatric* or paediatric* or peadiatric* or prematur* or preterm*).mp. |
| 16 | school*.ti,ab. |
| 17 | or/14-16^[[1]](#footnote-1)^ |
| 18 | randomized controlled trial.pt. |
| 19 | controlled clinical trial.pt. |
| 20 | randomized.ab. |
| 21 | placebo.ab. |
| 22 | clinical trials as topic.sh. |
| 23 | randomly.ab. |
| 24 | trial.ti.^[[2]](#footnote-2)^ |
| 25 | Epidemiologic studies/ |
| 26 | exp case control studies/ |
| 27 | exp cohort studies/ |
| 28 | Case control.tw. |
| 29 | (cohort adj (study or studies)).tw. |
| 30 | Cohort analy$.tw. |
| 31 | (Follow up adj (study or studies)).tw. |
| 32 | (observational adj (study or studies)).tw. |
| 33 | Longitudinal.tw. |
| 34 | Retrospective.tw. |
| 35 | Cross sectional.tw. |
| 36 | Cross-sectional studies/ |
| 37 | or/18-36^[[3]](#footnote-3)^ |
| 38 | exp animals/ not humans.sh. |
| 39 | 37 not 38 |
| 40 | 8 and 13 and 17 and 39 |

1. Pediatric search filter adapted from: Leclercq E, Leeflang MM, van Dalen EC, Kremer LC. Validation of search filters for identifying pediatric studies in PubMed. J Pediatr. 2013 Mar;162(3):629-634.e2. [↑](#footnote-ref-1)
2. Cochrane Highly Sensitive Search Strategy for identifying randomized trials in MEDLINE: sensitivity-maximizing version (2008 revision); Ovid format (Box 6.4.c). In Higgins JPT, Green S (editors). *Cochrane Handbook for Systematic Reviews of Interventions* Version 5.1.0 [updated March 2011]. The Cochrane Collaboration, 2011. Available from www.handbook.cochrane.org. [↑](#footnote-ref-2)
3. Observational studies filter, Scottish Intercollegiate Guidelines Network. Available from https://www.sign.ac.uk/search-filters.html. [↑](#footnote-ref-3)
