# Supplementary material for: Probiotics for the prevention of antibiotic-associated adverse events in children—A scoping review to inform development of a core outcome set
Source: PLoS One. 2020 May 29;15(5):e0228824. doi: 10.1371/journal.pone.0228824 (PMC7259577; doi:10.1371/journal.pone.0228824)
Supplement: S2 Table — (DOCX) [file pone.0228824.s003.docx]

**S2 Table.** Excluded studies with reasons of exclusion

| **Study ID** | **Reason for exclusion** |
| --- | --- |
| Ameen 2019 [1] | Non-eligible population |
| Andaloro 2019 [2] | Outcomes of interest not reported |
| Awasthi 2000 [3] | Commentary on another study |
| Cherian 2012 [4] | Non-eligible population  Outcomes of interest not reported |
| Conway 2007 [5] | Non-eligible population |
| Czerwionka-Szaflarska 2006 [6] | Article not in English |
| Dajani 2013 [7] | Non-eligible population |
| Dajani 2013 [8] | Non-eligible population |
| De Bortoli 2007 [9] | Non-eligible population |
| Doyle 2018 [10] | Outcomes of interest not reported |
| Duman 2005 [11] | Non-eligible population |
| Francavilla 2008 [12] | Non-eligible population |
| Francavilla 2014 [13] | Non-eligible population |
| Goldman 2006 [14] | Outcomes of interest not reported |
| Huang 2011 [15] | Conference abstract |
| Islek 2015 [16] | Not a probiotic intervention |
| Kim 2008 [17] | Non-eligible population |
| Kitz 2012 [18] | Outcomes of interest not reported |
| Korpela 2018 [19] | Outcomes of interest not reported |
| Kumar 2013 [20] | Non-eligible population |
| Li 2008 [21] | Secondary study |
| Li 2018 [22] | Outcomes of interest not reported |
| Li 2019 [23] | Outcomes of interest not reported |
| Lukasik 2018 [24] | Study protocol |
| Madden Fuentes 2015 [25] | Outcomes of interest not reported |
| Maziade 2013 [26] | Non-eligible population |
| Mohseni 2013 [27] | Outcomes of interest not reported |
| Murphy 2016 [28] | Not a probiotic intervention |
| Namkin 2016 [29] | Non-eligible population |
| Nista 2004 [30] | Non-eligible population |
| Pantoflickova 2003 [31] | Non-eligible population |
| Prado 1980 [32] | Article not in English |
| Rohrenbach 2009 [33] | Article not in English |
| Saneeyan 2011 [34] | Article not in English |
| Schrezenmeir 2004 [35] | Not a probiotic intervention |
| Sirvan 2017 [36] | Not a probiotic intervention |
| Song 2010 [37] | Non-eligible population |
| Srinivasan 2006 [38] | Non-eligible population |
| Tamma 2017 [39] | Non-eligible population |
| Tongtawee 2016 [40] | Non-eligible population |
| Uitz 2017 [41] | Non-eligible population |
| Ustundag 2017 [42] | Not a probiotic intervention |
| Valsecchi 2014 [43] | Outcomes of interest not reported |
| Wan 2017 [44] | Article not in English |
| Wang 2017 [45] | Non-eligible population |
| Witsell 1995 [46] | Non-eligible population |
| Xiang 2019 [47] | Non-eligible population and intervention |
| Zhao 2014 [48] | Article not in English |
| Zheng 2012 [49] | Article not in English |
| Ziemniak 2006 [50] | Non-eligible population |

1. Ameen AM, Abdulridha MK, Najeeb AA. Comparative effectiveness of probiotics timing regimen in helicobacter pylori-induced peptic ulcer disease patients. Journal of Pharmaceutical Sciences and Research. 2019;11(1):75-83.

2. Andaloro C, Santagati M, Stefani S, La Mantia I. Bacteriotherapy with Streptococcus salivarius 24SMB and Streptococcus oralis 89a oral spray for children with recurrent streptococcal pharyngotonsillitis: a randomized placebo-controlled clinical study. European archives of oto-rhino-laryngology : official journal of the European Federation of Oto-Rhino-Laryngological Societies (EUFOS) : affiliated with the German Society for Oto-Rhino-Laryngology - Head and Neck Surgery. 2019;276(3):879-87. doi: <https://dx.doi.org/10.1007/s00405-019-05346-3>.

3. Awasthi S. Lactobacillus GG reduced diarrhoea incidence in children treated with antibiotics. Evidence-Based Medicine. 2000;5(4):113. doi: 10.1136/ebm.5.4.113.

4. Cherian S, Sibyjoseph, Anitha S. Study of the prescribing pattern of probiotics in paediatric patients of a teritiary care teaching hospital, South India. International Journal of Pharmacy and Pharmaceutical Sciences. 2012;4(1):505-8.

5. Conway S, Hart A, Clark A, Harvey I. Does eating yogurt prevent antibiotic-associated diarrhoea? A placebo-controlled randomised controlled trial in general practice. The British journal of general practice : the journal of the Royal College of General Practitioners. 2007;57(545):953-9. doi: <https://dx.doi.org/10.3399/096016407782604811>.

6. Czerwionka-Szaflarska M, Kuczynska R, Mierzwa G, Bala G, Murawska S. Effect of probiotic bacteria supplementation on the tolerance of Helicobacter pylori eradication therapy in children and youth. Pediatria polska. 2006;81(5):334‐41. PubMed PMID: CN-00623129.

7. Dajani A, Hammour AA, Nounou ME, Zakaria M. Treatment of helicobacter pylori: Role of probiotics, an experience from the UAE. Journal of Gastroenterology and Hepatology. 2013;28:330-1. doi: 10.1111/jgh.12363_2.

8. Dajani AI, Abu Hammour AM, Yang DH, Chung PC, Nounou MA, Yuan KY, et al. Do probiotics improve eradication response to Helicobacter pylori on standard triple or sequential therapy? Saudi journal of gastroenterology : official journal of the Saudi Gastroenterology Association. 2013;19(3):113-20. doi: <https://dx.doi.org/10.4103/1319-3767.111953>.

9. De Bortoli N, Leonardi G, Ciancia E, Merlo A, Bellini M, Costa F, et al. Helicobacter pylori eradication: A randomized prospective study of triple therapy versus triple therapy plus lactoferrin and probiotics. American Journal of Gastroenterology. 2007;102(5):951-6. doi: 10.1111/j.1572-0241.2007.01085.x.

10. Doyle H, Pierse N, Tiatia R, Williamson D, Baker M, Crane J. Effect of Oral Probiotic Streptococcus salivarius K12 on Group A Streptococcus Pharyngitis: A Pragmatic Trial in Schools. The Pediatric infectious disease journal. 2018;37(7):619-23. doi: <https://dx.doi.org/10.1097/INF.0000000000001847>.

11. Duman DG, Bor S, Ozütemiz O, Sahin T, Oğuz D, Iştan F, et al. Efficacy and safety of Saccharomyces boulardii in prevention of antibiotic-associated diarrhoea due to Helicobacterpylori eradication. European journal of gastroenterology & hepatology. 2005;17(12):1357‐61. PubMed PMID: CN-00561517.

12. Francavilla R, Lionetti E, Castellaneta SP, Magistà AM, Maurogiovanni G, Bucci N, et al. Inhibition of Helicobacter pylori infection in humans by Lactobacillus reuteri ATCC 55730 and effect on eradication therapy: A pilot study. Helicobacter. 2008;13(2):127-34. doi: 10.1111/j.1523-5378.2008.00593.x.

13. Francavilla R, Polimeno L, Demichina A, Maurogiovanni G, Principi B, Scaccianoce G, et al. Lactobacillus reuteri strain combination in Helicobacter pylori infection: a randomized, double-blind, placebo-controlled study. Journal of clinical gastroenterology. 2014;48(5):407-13. doi: <https://dx.doi.org/10.1097/MCG.0000000000000007>.

14. Goldman CG, Barrado DA, Balcarce N, Rua EC, Oshiro M, Calcagno ML, et al. Effect of a probiotic food as an adjuvant to triple therapy for eradication of Helicobacter pylori infection in children. Nutrition (burbank, los angeles county, calif). 2006;22(10):984‐8. doi: 10.1016/j.nut.2006.06.008. PubMed PMID: CN-00572240.

15. Huang Y, Wang YH, Leung YK. Combination of Lactobacillus acidophilus and bifidobacillus with standard triple therapy (PPI plus amoxicillin and clarithromycin, STT) in the management of H. pylori infection: Therapeutic efficacy and changes in intestinal bacterial flora. Journal of Gastroenterology and Hepatology. 2011;26:256. doi: 10.1111/j.1440-1746.2011.06898.x.

16. Islek A, Sayar E, Yilmaz A, Artan R. Bifidobacterium lactis B94 plus inulin for Treatment of Helicobacter pylori infection in children: does it increase eradication rate and patient compliance? Acta gastro-enterologica Belgica. 2015;78(3):282-6.

17. Kim MN, Kim N, Lee SH, Park YS, Hwang JH, Kim JW, et al. The effects of probiotics on PPI-triple therapy for Helicobacter pylori eradication. Helicobacter. 2008;13(4):261‐8. doi: 10.1111/j.1523-5378.2008.00601.x. PubMed PMID: CN-00650099.

18. Kitz R, Martens U, Zieseniß E, Enck P, Rose MA. Probiotic E.faecalis - Adjuvant therapy in children with recurrent rhinosinusitis. Central European Journal of Medicine. 2012;7(3):362-5. doi: 10.2478/s11536-011-0160-8.

19. Korpela K, Salonen A, Vepsalainen O, Suomalainen M, Kolmeder C, Varjosalo M, et al. Probiotic supplementation restores normal microbiota composition and function in antibiotic-treated and in caesarean-born infants. Microbiome. 2018;6(1):182. doi: <https://dx.doi.org/10.1186/s40168-018-0567-4>.

20. Kumar S, Bansal A, Chakrabarti A, Singhi S. Evaluation of efficacy of probiotics in prevention of candida colonization in a PICU-a randomized controlled trial. Critical care medicine. 2013;41(2):565-72. doi: <https://dx.doi.org/10.1097/CCM.0b013e31826a409c>.

21. Li N, Zheng B, Cai HF, Chen YH, Qiu MQ, Liu MB. Cost-effectiveness analysis of oral probiotics for the prevention of Clostridium difficile-associated diarrhoea in children and adolescents. Journal of Hospital Infection. 2018;99(4):469-74. doi: 10.1016/j.jhin.2018.04.013.

22. Li B, Zheng J, Zhang X, Hong S. Probiotic Lactobacillus casei Shirota improves efficacy of amoxicillin-sulbactam against childhood fast breathing pneumonia in a randomized placebo-controlled double blind clinical study. Journal of clinical biochemistry and nutrition. 2018;63(3):233-7. doi: <https://dx.doi.org/10.3164/jcbn.17-117>.

23. Li KL, Wang BZ, Li ZP, Li YL, Liang JJ. Alterations of intestinal flora and the effects of probiotics in children with recurrent respiratory tract infection. World journal of pediatrics : WJP. 2019;15(3):255-61. doi: <https://dx.doi.org/10.1007/s12519-019-00248-0>.

24. Lukasik J, Szajewska H. Effect of a multispecies probiotic on reducing the incidence of antibiotic-associated diarrhoea in children: a protocol for a randomised controlled trial. BMJ open. 2018;8(5):e021214. doi: <https://dx.doi.org/10.1136/bmjopen-2017-021214>.

25. Madden-Fuentes RJ, Arshad M, Ross SS, Seed PC. Efficacy of Fluoroquinolone/Probiotic Combination Therapy for Recurrent Urinary Tract Infection in Children: A Retrospective Analysis. Clinical therapeutics. 2015;37(9):2143-7. doi: <https://dx.doi.org/10.1016/j.clinthera.2015.06.018>.

26. Maziade PJ, Andriessen JA, Pereira P, Currie B, Goldstein EJC. Impact of adding prophylactic probiotics to a bundle of standard preventative measures for Clostridium difficile infections: enhanced and sustained decrease in the incidence and severity of infection at a community hospital. Current medical research and opinion. 2013;29(10):1341-7. doi: <https://dx.doi.org/10.1185/03007995.2013.833501>.

27. Mohseni MJ, Aryan Z, Emamzadeh-Fard S, Paydary K, Mofid V, Joudaki H, et al. Combination of probiotics and antibiotics in the prevention of recurrent urinary tract infection in children. Iranian journal of pediatrics. 2013;23(4):430‐8. PubMed PMID: CN-00918907.

28. Murphy JL, Fenn N, Pyle L, Heizer H, Hughes S, Nomura Y, et al. Adverse events in pediatric patients receiving long-term oral and intravenous antibiotics. Hospital Pediatrics. 2016;6(6):330-8. doi: 10.1542/hpeds.2015-0069.

29. Namkin K, Zardast M, Basirinejad F. Saccharomyces Boulardii in Helicobacter Pylori Eradication in Children: A Randomized Trial From Iran. Iranian journal of pediatrics. 2016;26(1):e3768. doi: <https://dx.doi.org/10.5812/ijp.3768>.

30. Nista EC, Candelli M, Cremonini F, Cazzato IA, Zocco MA, Franceschi F, et al. Bacillus clausii therapy to reduce side-effects of anti-Helicobacter pylori treatment: randomized, double-blind, placebo controlled trial. Alimentary pharmacology & therapeutics. 2004;20(10):1181-8.

31. Pantoflickova D, Corthesy-Theulaz I, Dorta G, Stolte M, Isler P, Rochat F, et al. Favourable effect of regular intake of fermented milk containing Lactobacillus johnsonii on Helicobacter pylori associated gastritis. Alimentary pharmacology & therapeutics. 2003;18(8):805-13.

32. Prado V, Agüero ME, Ernst Y, Marín P, Díaz MC. Effects of administration of lactobacilli on intestinal flora in infants treated with broad spectrum antibiotics. Rev Chil Pediatr. 1980;51(1):9‐12. PubMed PMID: CN-01623988.

33. Röhrenbach J, Matthess A, Maier R, Von Bünau R. Treatment of children with E. coli strain Nissle 1917. Results of a prospective data collection with 668 patients. Padiatrische Praxis. 2009;73(4):645-52.

34. Saneeyan H, Layegh S, Rahimi H. Effectivness of probiotic on treatment of Helicobacter pylori infection in children. Journal of isfahan medical school. 2011;29(146):882‐9. PubMed PMID: CN-00893921.

35. Schrezenmeir J, Heller K, McCue M, Llamas C, Lam W, Burow H, et al. Benefits of oral supplementation with and without synbiotics in young children with acute bacterial infections. Clinical pediatrics. 2004;43(3):239-49.

36. N Sirvan B, K Usta M, U Kizilkan N, Urganci N. Are Synbiotics added to the Standard Therapy to eradicate Helicobacter pylori in Children Beneficial? A Randomized Controlled Study. Euroasian journal of hepato-gastroenterology. 2017;7(1):17-22. doi: <https://dx.doi.org/10.5005/jp-journals-10018-1205>.

37. Song MJ, Park DI, Park JH, Kim HJ, Cho YK, Sohn CI, et al. The effect of probiotics and mucoprotective agents on PPI-based triple therapy for eradication of helicobacter pylori. Helicobacter. 2010;15(3):206-13. doi: 10.1111/j.1523-5378.2010.00751.x.

38. Srinivasan R, Meyer R, Padmanabhan R, Britto J. Clinical safety of Lactobacillus casei shirota as a probiotic in critically ill children. Journal of pediatric gastroenterology and nutrition. 2006;42(2):171-3.

39. Tamma PD, Avdic E, Li DX, Dzintars K, Cosgrove SE. Association of adverse events with antibiotic use in hospitalized patients. JAMA Internal Medicine. 2017;177(9):1308-15. doi: 10.1001/jamainternmed.2017.1938.

40. Tongtawee T, Dechsukhum C, Leeanansaksiri W, Kaewpitoon S, Kaewpitoon N, Loyd RA, et al. Effect of Pretreatment with Lactobacillus delbrueckii and Streptococcus thermophillus on Tailored Triple Therapy for Helicobacter pylori Eradication: A Prospective Randomized Controlled Clinical Trial. Asian Pacific journal of cancer prevention : APJCP. 2015;16(12):4885-90.

41. Uitz E, Tonninger-Bahadori K, Nekrep K, Bahadori B. The effect of lactobacillus casei rhamnosus (lcr35) supplementation on the adherence, tolerance and efficacy of helicobacter pylori eradication therapy: An open-label, observational, non-interventional, multicentre pilot study. International Journal of Probiotics and Prebiotics. 2017;12(4):159-66.

42. Ustundag GH, Altuntas H, Soysal YD, Kokturk F. The Effects of Synbiotic "Bifidobacterium lactis B94 plus Inulin" Addition on Standard Triple Therapy of Helicobacter pylori Eradication in Children. Canadian journal of gastroenterology & hepatology. 2017;2017:8130596. doi: <https://dx.doi.org/10.1155/2017/8130596>.

43. Valsecchi C, Marseglia A, Montagna L, Tagliacarne SC, Elli M, Licari A, et al. Evaluation of the effects of a probiotic supplementation with respect to placebo on intestinal microflora and secretory IgA production, during antibiotic therapy, in children affected by recurrent airway infections and skin symptoms. Journal of biological regulators and homeostatic agents. 2014;28(1):117-24.

44. Wan CM, Yu H, Liu G, Xu HM, Mao ZQ, Xu Y, et al. A multicenter randomized controlled study of Saccharomyces boulardii in the prevention of antibiotic-associated diarrhea in infants and young children. Zhonghua er ke za zhi = Chinese journal of pediatrics. 2017;55(5):349-54. doi: <https://dx.doi.org/10.3760/cma.j.issn.0578-1310.2017.05.008>.

45. Wang ZJ, Chen XF, Zhang ZX, Li YC, Deng J, Tu J, et al. Effects of anti-Helicobacter pylori concomitant therapy and probiotic supplementation on the throat and gut microbiota in humans. Microbial pathogenesis. 2017;109:156‐61. doi: 10.1016/j.micpath.2017.05.035. PubMed PMID: CN-01454307.

46. Witsell DL, Garrett CG, Yarbrough WG, Dorrestein SP, Drake AF, Weissler MC. Effect of Lactobacillus acidophilus on antibiotic-associated gastrointestinal morbidity: a prospective randomized trial. The Journal of otolaryngology. 1995;24(4):230-3.

47. Xiang R, Tang Q, Chen XQ, Li MY, Yang MX, Yun X, et al. Effects of Zinc Combined with Probiotics on Antibiotic-associated Diarrhea Secondary to Childhood Pneumonia. Journal of tropical pediatrics. 2019;65(5):421-6. doi: <https://dx.doi.org/10.1093/tropej/fmy069>.

48. Zhao HM, Ou-Yang HJ, Duan BP, Xu B, Chen ZY, Tang J, et al. Clinical effect of triple therapy combined with Saccharomyces boulardii in the treatment of Helicobacter pylori infection in children. Zhongguo dang dai er ke za zhi [Chinese journal of contemporary pediatrics]. 2014;16(3):230‐3. PubMed PMID: CN-01117599.

49. Investigating Group for Prevention of AADiCwPbCB, Bifidobacterium. Multicenter, randomized, controlled clinical trial on preventing antibiotic-associated diarrhea in children with pneumonia using the live Clostridium butyricum and Bifidobacterium combined Powder. Zhonghua er ke za zhi = Chinese journal of pediatrics. 2012;50(10):732-6.

50. Ziemniak W. Efficacy of Helicobacter pylori eradication taking into account its resistance to antibiotics. Journal of physiology and pharmacology : an official journal of the Polish Physiological Society. 2006;57 Suppl 3:123-41.
