# Supplementary material for: Probiotics for the prevention of antibiotic-associated adverse events in children—A scoping review to inform development of a core outcome set
Source: PLoS One. 2020 May 29;15(5):e0228824. doi: 10.1371/journal.pone.0228824 (PMC7259577; doi:10.1371/journal.pone.0228824)
Supplement: S3 Table — (DOCX) [file pone.0228824.s004.docx]

**S3 Table.** Characteristics of the included studies

| Study ID | Study  type | Setting | Number and age of participants | Indication for antibiotic therapy | Types of antibiotics | Intervention | Control |
| --- | --- | --- | --- | --- | --- | --- | --- |
| Ahmad 2013 [1] | RCT | Outpatient | N = 66  Age: 3-14 y | H. pylori infection | Amoxicillin + furazolidone | *L. acidophilus, L. rhamnosus, L. bulgaricus, L. casei, S. thermophilus, B. infantis and B. breve* | Placebo |
| Akcam 2015 [2] | RCT | Outpatient | N = 61  Age: 7-18 y | H. pylori infection | Amoxicillin + clarithromycin | *L. casei 2401, L. acidophilus 2027 and B. lactis 2211* | No treatment |
| Arvola 1999 [3] | RCT | Mixed setting | N = 119  Age: 2 – 11,8 y | Acute respiratory infections | Oral penicillin, amoxicillin, cephalosporins, erythromycin or trimethoprim-sulphamethoxazole | *Lactobacillus GG* | Placebo |
| Basnet 2017 [4] | NRT | Outpatient | N = 174  Age: 6 m-16 y | Respiratory tract infections | Amoxicillin + clavulanic acid | *L. sporogenes, St. faecalis, C. butyricum, Ba. mesentericus TO-A 1* | No treatment |
| Bau 2020 [5] | Cohort study | Mixed setting | N = 336  Age: 1m – 17 y | Any indication | Any antibiotics | Any probiotics, according to patient choice (observational study), most commonly: *L. rhamnosus GG, L. reuteri DSM 17938* | No treatment |
| Bin 2015 [6] | RCT | Probably outpatient | N = 194  Age: 22 m – 16 y | H. pylori infection | Amoxicillin+clarithromycin, metronidazole | *S. boulardii CNCM I-745* | No treatment |
| Correa 2005 [7] | RCT | Inpatient | N = 169  Age: 6-36 m | Not described | Penicillin, ampicillin, oxacillin, amoxicillin, cephalosporin, amoxicillin+clavulanic acid, others | *B. lactis and St. thermophilus* | Placebo |
| Dharani 2017  [8] | RCT | Outpatient | N=100  Age: 1-15 y | Impetigo | Azithromycin | *L. sporegens, S. faecalis, Clostridium butyricum and Bacillus mesentericus* | No treatment |
| Erdeve 2004 [9] | NRT | Setting unclear | N = 653  Age: 1-15 y | Not described | Sulbactam-ampicillin or azithromycin | *S. boulardii* | No treatment |
| Esposito 2018 [10] | RCT | Inpatient | N = 90  Age: 11-36 m | UTI prophylaxis after hypospadias repair | Amoxicillin – clavulanate or macrolide | *L. rhamnosus GG* | Placebo |
| Fox 2015 [11] | RCT | Outpatient | N = 72  Age: 1-12 y | Any indication other than prophylaxis | Beta-lactams, macrolides, tetracyclines, | *L. rhamnosus GG, B. lactis BB-12, L. acidophilus La-5* | Placebo |
| Georgieva 2015 [12] | RCT | Inpatient | N = 100  Age: 3-12 y | Any infection | Aminoglycosides, fluoroquinolones, beta-lactams, metronidazole | *L. reuteri 17938* | Placebo |
| Hurduc 2009 [13] | RCT | Probably outpatient | N = 90  Age: 3-18 y | H. pylori infection | Amoxicillin + clarithromycin | *S. boulardii* | No treatment |
| Jindal 2017 [14] | RCT | Outpatient | N = 600  Age: 6 m – 12 y | UTI, otitis media, tonsillitis | Beta-lactams | *S. boulardii* | No treatment |
| Jirapinyo 2002 [15] | RCT | Inpatient | N = 18  Age: 1-36 m | Sepsis, meningitis | Broad-spectrum antibiotics | *L. acidophilus, B. infantis* | Placebo |
| Kołodziej 2018 [16] | RCT | Inpatient | N = 250  Age: 0-18 y | Any infection | Any antibiotics | *L. reuteri 17938* | Placebo |
| Korpela 2016 [17] | RCT | Outpatient | N = 231  Age: 2-6 y | Infections | Macrolides, penicillins, cephalosporins, trimethoprim-sulphamethoxazole | *L. rhamnosus GG* | Placebo |
| Kotowska 2005 [18] | RCT | Mixed setting | N = 269  Age: 6 – 14 y | Otitis media, respiratory tract infection | Any antibiotics | *S. boulardii* | Placebo |
| Lionetti 2006 [19] | RCT | Probably outpatient | N =40  Age: 3,3-18 y | H. pylori infection | Amoxicillin + clarithromycin | *L. reuteri ATCC 55730* | Placebo |
| Merenstein 2009 [20] | RCT | Outpatient | N = 125  Age: 1-5 y | Upper respiratory infections | Not described | *Kefir containing various strains of bacteria.* | Placebo |
| Okazaki 2016 [21] | RCT | Inpatient | N = 30  Age: <15 y | Postoperative prophylaxis | Not described | *B. Breve strain Yakult* | Placebo |
| Olek 2017 [22] | RCT | Outpatient | N = 447  Age: 1-11 y | Common infections | Beta-lactams, trimethoprim-sulphamethoxazole, macrolides | *L. plantarum DSM9843* | Placebo |
| Plewińska 2006 [23] | NRT | Outpatient | N = 60  Age: 8,8-18,3 y | H. pylori infection | Amoxicillin, clarithromycin | *L. acidophilus R0052, L. rhamnosus R0011* | Placebo |
| Ranasinghe 2008 [24] | RCT | Inpatient | N = 76  Age: 6 m – 5 y | Any indication other than diarrhea | Amoxicillin, amoxiclav | *Yogurt containing bifidobacteria* | No treatment |
| Ruszczyński 2008 [25] | RCT | Mixed setting | N = 240  Age: 3m-14 y | Common infections | Penicillins, broad-spectrum penicillins (ampicillin, amoxicillin, amoxicillin plus clavulanate), cephalosporins, macrolides, clindamycin | *L. rhamnosus E/N, oxy, pen* | Placebo |
| Seki 2003 [26] | NRT | Mixed setting | N = 110  Age: 1 m - 15 y | Upper respiratory infections, gastroenteritis | Penicillin, cephalosporin, tetracycline, fosfomycin | *C. butyricum Miyairi* | No treatment |
| Shahraki 2017 [27] | RCT | Outpatient | N = 50  Age: 5-18 y | H. pylori infection | Amoxicillin + clarithromycin | *L. reuteri* | No treatment |
| Shan 2013 [28] | RCT | Inpatient | N = 333  Age: 6-14 y | Lower respiratory tract infection | Cefepime, Cefoperazone+sulbactam, Cefuroxime, Amoxicillin+clavulanic acid, Erythromycin, others | *S. boulardii* | No treatment |
| Sykora 2005 [29] | RCT | Probably outpatient | N = 86  Mean age: 12.6y | H. pylori infection | Amoxicillin, clarithromycin | *L .casei DN-114 001* | Placebo |
| Szajewska 2009 [30] | RCT | Outpatient | N = 66  Age: 5-17 y | H. pylori infection | Clarithromycin | *L. rhamnosus GG* | Placebo |
| Szymański 2008 [31] | RCT | Mixed setting | N = 78  Age: 5 m – 16 y | Otitis media, and/or respiratory tract infections, and/or urinary tract infections | Amoxicillin, amoxicillin+clavulanate, cephalosporins, penicillin, macrolides, aminoglycosides | *B. longum PL03, L. rhamnosus KL53A and L. plantarum PL02* | Placebo |
| Tankanow 1990 [32] | RCT | Outpatient | N = 60  Age: 5m-6y | Not described precisely, mostly otitis media and pharyngitis | Amoxicillin | *L. acidophilus and L. bulgaricus* | Placebo |
| Tolone 2012 [33] | RCT | Probably outpatient | N = 68  Age mean: 8.3 y | H. pylori infection | Amoxicillin + clarithromycin | *L. plantarum, L. reuteri, L. casei subsp. rhamnosus, B. infantis and B. longum, L. salivarius, L. acidophilus,*  *S. thermophilus, L. sporogenes.* | No treatment |
| Vanderhoof 1999 [34] | RCT | Outpatient | N = 202  Age: 6 m – 10 y | Upper to lower respiratory tract; urinary tract; soft tissues infection; skin infection | Oral antibiotics | *L. rhamnosus GG* | Placebo |
| Wang 2014 [35] | RCT | Probably outpatient | N = 88  Age mean: 7.8 y | H. pylori infection | Clarithromycin + amoxicillin/metronidazole | *L. acidophilus-5 and B. biﬁdum-12* | No treatment |
| Zakordonets 2016 [36] | RCT | Inpatient | N = 40  Age: 3-17 y | Moderate or severe acute bacterial infection diseases | Ceftriaxone | *Lactobacilli and Lactococci, Bifidobacteria, propionate-oxidising bacteria, acetic acid bacteria* | No treatment |
| Zoppi 2001 [37] | RCT | Inpatient | N = 51  Age: mean 5,1 y | Febrile respiratory tract infections | Ceftriaxone | *7 experimental arms: S. boulardii (1); E. species (2); lactulose (3); L. casei GG (4) L. rhamnosus, L. bifidus, and L. acidophilus (5); B. bifidum and L. acidophilus (6); or a mixture of various lactobacilli and bifidobacteria at high concentrations (7).* | No treatment |

1. Ahmad K, Fatemeh F, Mehri N, Maryam S. Probiotics for the treatment of pediatric helicobacter pylori infection: a randomized double blind clinical trial. Iranian journal of pediatrics. 2013;23(1):79-84.

2. Akcam M, Koca T, Salman H, Karahan N. The effects of probiotics on treatment of Helicobacter pylori eradication in children. Saudi medical journal. 2015;36(3):286-90. doi: <https://dx.doi.org/10.15537/smj.2015.3.10124>.

3. Arvola T, Laiho K, Torkkeli S, Mykkanen H, Salminen S, Maunula L, et al. Prophylactic Lactobacillus GG reduces antibiotic-associated diarrhea in children with respiratory infections: a randomized study. Pediatrics. 1999;104(5):e64.

4. Basnet S, Gauchan E, Adhikari S, Sathian B. Probiotics in the prevention of antibiotic associated diarrhoea in a tertiary teaching hospital in pokhara: A prospective study. Journal of Clinical and Diagnostic Research. 2017;11(10):SC11-SC3. doi: 10.7860/JCDR/2017/25936.10777.

5. Baù M, Moretti A, Bertoni E, Vazzoler V, Luini C, Agosti M. Risk and Protective Factors for Gastrointestinal Symptoms associated with Antibiotic Treatment in Children: A Population Study. Pediatric Gastroenterology, Hepatology & Nutrition. 2020;23:35. doi: 10.5223/pghn.2020.23.1.35.

6. Bin Z, Ya-Zheng X, Zhao-Hui D, Bo C, Li-Rong J, Vandenplas Y. The Efficacy of Saccharomyces boulardii CNCM I-745 in Addition to Standard Helicobacter pylori Eradication Treatment in Children. Pediatric gastroenterology, hepatology & nutrition. 2015;18(1):17-22. doi: <https://dx.doi.org/10.5223/pghn.2015.18.1.17>.

7. Corrêa NB, Péret Filho LA, Penna FJ, Lima FM, Nicoli JR. A randomized formula controlled trial of Bifidobacterium lactis and Streptococcus thermophilus for prevention of antibiotic-associated diarrhea in infants. Journal of clinical gastroenterology. 2005;39(5):385‐9. PubMed PMID: CN-00521370.

8. Dharani Sudha G, Nirmala P, Ramanathan R, Samuel V. Comparative study of efficacy and safety of azithromycin alone and in combination with probiotic in the treatment of impetigo in children. International Journal of Current Pharmaceutical Research. 2017;9(6):52-5. doi: 10.22159/ijcpr.2017v9i6.23429.

9. Erdeve O, Tiras U, Dallar Y. The probiotic effect of Saccharomyces boulardii in a pediatric age group. Journal of tropical pediatrics. 2004;50(4):234-6. doi: <https://dx.doi.org/10.1093/tropej/50.4.234>.

10. Esposito C, Roberti A, Turra F, Cerulo M, Severino G, Settimi A, et al. Frequency of Antibiotic-Associated Diarrhea and Related Complications in Pediatric Patients Who Underwent Hypospadias Repair: a Comparative Study Using Probiotics vs Placebo. Probiotics and antimicrobial proteins. 2018;10(2):323-8. doi: <https://dx.doi.org/10.1007/s12602-017-9324-4>.

11. Fox MJ, Ahuja KD, Robertson IK, Ball MJ, Eri RD. Can probiotic yogurt prevent diarrhoea in children on antibiotics? A double-blind, randomised, placebo-controlled study. BMJ open. 2015;5(1):e006474. doi: 10.1136/bmjopen-2014-006474. PubMed PMID: CN-01111087.

12. Georgieva M, Pancheva R, Rasheva N, Usheva N, Ivanova L, Koleva K. Use of the probiotic Lactobacillus reuteri DSM 17938 in the prevention of antibioticassociated infections in hospitalized bulgarian children: a randomized, controlled trial. Journal of IMAB - annual proceeding (scientific papers). 2015;21(4):895‐900. doi: 10.5272/jimab.2015214.895. PubMed PMID: CN-01133218.

13. Hurduc V, Plesca D, Dragomir D, Sajin M, Vandenplas Y. A randomized, open trial evaluating the effect of Saccharomyces boulardii on the eradication rate of Helicobacter pylori infection in children. Acta paediatrica (Oslo, Norway : 1992). 2009;98(1):127-31. doi: <https://dx.doi.org/10.1111/j.1651-2227.2008.00977.x>.

14. Jindal M, Goyal Y, Lata S, Sharma RK. Preventive role of probiotic in antibiotic associated diarrhoea in children. Indian Journal of Public Health Research and Development. 2017;8(3):66-9. doi: 10.5958/0976-5506.2017.00162.0.

15. Jirapinyo P, Densupsoontorn N, Thamonsiri N, Wongarn R. Prevention of antibiotic-associated diarrhea in infants by probiotics. Journal of the Medical Association of Thailand = Chotmaihet thangphaet. 2002;85 Suppl 2:S739-42.

16. Kolodziej M, Szajewska H. Lactobacillus reuteri DSM 17938 in the prevention of antibiotic-associated diarrhoea in children: a randomized clinical trial. Clinical microbiology and infection : the official publication of the European Society of Clinical Microbiology and Infectious Diseases. 2018. doi: <https://dx.doi.org/10.1016/j.cmi.2018.08.017>.

17. Korpela K, Salonen A, Virta LJ, Kumpu M, Kekkonen RA, de Vos WM. Lactobacillus rhamnosus GG Intake Modifies Preschool Children's Intestinal Microbiota, Alleviates Penicillin-Associated Changes, and Reduces Antibiotic Use. PloS one. 2016;11(4):e0154012. doi: <https://dx.doi.org/10.1371/journal.pone.0154012>.

18. Kotowska M, Albrecht P, Szajewska H. Saccharomyces boulardii in the prevention of antibiotic-associated diarrhoea in children: a randomized double-blind placebo-controlled trial. Alimentary pharmacology & therapeutics. 2005;21(5):583-90.

19. Lionetti E, Miniello VL, Castellaneta SP, Magista AM, de Canio A, Maurogiovanni G, et al. Lactobacillus reuteri therapy to reduce side-effects during anti-Helicobacter pylori treatment in children: a randomized placebo controlled trial. Alimentary pharmacology & therapeutics. 2006;24(10):1461-8.

20. Merenstein DJ, Foster J, D'Amico F. A randomized clinical trial measuring the influence of kefir on antibiotic-associated diarrhea: the measuring the influence of Kefir (MILK) Study. Archives of pediatrics & adolescent medicine. 2009;163(8):750-4. doi: <https://dx.doi.org/10.1001/archpediatrics.2009.119>.

21. Okazaki T, Asahara T, Yamataka A, Ogasawara Y, Lane GJ, Nomoto K, et al. Intestinal Microbiota in Pediatric Surgical Cases Administered Bifidobacterium Breve: a Randomized Controlled Trial. Journal of pediatric gastroenterology and nutrition. 2016;63(1):46‐50. doi: 10.1097/mpg.0000000000001140. PubMed PMID: CN-01165832.

22. Olek A, Woynarowski M, Ahren IL, Kierkus J, Socha P, Larsson N, et al. Efficacy and Safety of Lactobacillus plantarum DSM 9843 (LP299V) in the Prevention of Antibiotic-Associated Gastrointestinal Symptoms in Children-Randomized, Double-Blind, Placebo-Controlled Study. The Journal of pediatrics. 2017;186:82-6. doi: <https://dx.doi.org/10.1016/j.jpeds.2017.03.047>.

23. Plewinska EM, Planeta-Malecka I, Bak-Romaniszyn L, Czkwianlanc E, Malecka-Panas E. Probiotics in the treatment of Helicobacter pylori infection in children. Gastroenterologia polska. 2006;13(4):315‐9. PubMed PMID: CN-00623178.

24. Ranasinghe J, Gamlath G, Samitha S, Abeygunawardena A. Prophylactic use of yoghurt reduces antibiotic induced diarrhoea in children. Sri Lanka Journal of Child Health. 2008;36(2):53-6. doi: <http://doi.org/10.4038/sljch.v36i2.50>.

25. Ruszczynski M, Radzikowski A, Szajewska H. Clinical trial: effectiveness of Lactobacillus rhamnosus (strains E/N, Oxy and Pen) in the prevention of antibiotic-associated diarrhoea in children. Alimentary pharmacology & therapeutics. 2008;28(1):154-61. doi: <https://dx.doi.org/10.1111/j.1365-2036.2008.03714.x>.

26. Seki H, Shiohara M, Matsumura T, Miyagawa N, Tanaka M, Komiyama A, et al. Prevention of antibiotic-associated diarrhea in children by Clostridium butyricum MIYAIRI. Pediatr Int. 2003;45(1):86-90. Epub 2003/03/26. PubMed PMID: 12654076.

27. Shahraki T, Shahraki M, Shahri ES, Mohammadi M. No significant impact of Lactobacillus reuteri on eradication of Helicobacter pylori in children (double-blind randomized clinical trial). Iranian red crescent medical journal. 2017;19(3) (no pagination). doi: 10.5812/ircmj.42101. PubMed PMID: CN-01366602.

28. Shan LS, Hou P, Wang ZJ, Liu FR, Chen N, Shu LH, et al. Prevention and treatment of diarrhoea with Saccharomyces boulardii in children with acute lower respiratory tract infections. Beneficial microbes. 2013;4(4):329‐34. doi: 10.3920/bm2013.0008. PubMed PMID: CN-00959577.

29. Sykora J, Valeckova K, Amlerova J, Siala K, Dedek P, Watkins S, et al. Effects of a specially designed fermented milk product containing probiotic Lactobacillus casei DN-114 001 and the eradication of H. pylori in children: a prospective randomized double-blind study. Journal of clinical gastroenterology. 2005;39(8):692-8.

30. Szajewska H, Albrecht P, Topczewska-Cabanek A. Randomized, double-blind, placebo-controlled trial: effect of lactobacillus GG supplementation on Helicobacter pylori eradication rates and side effects during treatment in children. Journal of pediatric gastroenterology and nutrition. 2009;48(4):431-6.

31. Szymanski H, Armanska M, Kowalska-Duplaga K, Szajewska H. Bifidobacterium longum PL03, Lactobacillus rhamnosus KL53A, and Lactobacillus plantarum PL02 in the prevention of antibiotic-associated diarrhea in children: a randomized controlled pilot trial. Digestion. 2008;78(1):13-7. doi: <https://dx.doi.org/10.1159/000151300>.

32. Tankanow RM, Ross MB, Ertel IJ, Dickinson DG, McCormick LS, Garfinkel JF. A double-blind, placebo-controlled study of the efficacy of Lactinex in the prophylaxis of amoxicillin-induced diarrhea. DICP : the annals of pharmacotherapy. 1990;24(4):382-4.

33. Tolone S, Pellino V, Vitaliti G, Lanzafame A, Tolone C. Evaluation of Helicobacter Pylori eradication in pediatric patients by triple therapy plus lactoferrin and probiotics compared to triple therapy alone. Italian journal of pediatrics. 2012;38:63. doi: <https://dx.doi.org/10.1186/1824-7288-38-63>.

34. Vanderhoof JA, Whitney DB, Antonson DL, Hanner TL, Lupo JV, Young RJ. Lactobacillus GG in the prevention of antibiotic-associated diarrhea in children. The Journal of pediatrics. 1999;135(5):564-8.

35. Wang YH, Huang Y. Effect of Lactobacillus acidophilus and Bifidobacterium bifidum supplementation to standard triple therapy on Helicobacter pylori eradication and dynamic changes in intestinal flora. World journal of microbiology & biotechnology. 2014;30(3):847‐53. doi: 10.1007/s11274-013-1490-2. PubMed PMID: CN-01014256.

36. Zakordonets L, Tolstanova G, Yankovskiy D, Dyment H, Kramarev S. Different regimes of multiprobiotic for prevention of immediate and delayed side effects of antibiotic therapy in children. Research journal of pharmaceutical, biological and chemical sciences. 2016;7(3):2194‐201. PubMed PMID: CN-01167212.

37. Zoppi G, Cinquetti M, Benini A, Bonamini E, Bertazzoni E. Modulation of the intestinal ecosystem by probiotics and lactulose in children during treatment with ceftriaxone. Current Therapeutic Research-clinical and Experimental - CURR THER RES. 2001;62:418-35. doi: 10.1016/S0011-393X(01)89006-8.
