# Supplementary material for: Probiotics for the prevention of antibiotic-associated adverse events in children—A scoping review to inform development of a core outcome set
Source: PLoS One. 2020 May 29;15(5):e0228824. doi: 10.1371/journal.pone.0228824 (PMC7259577; doi:10.1371/journal.pone.0228824)
Supplement: S4 Table — (DOCX) [file pone.0228824.s005.docx]

**S4 Table.** Outcomes identified in the included studies.

|  | Core area | Pathophysiological manifestations | | | | | | | | | | | | | | | | | | | | | | | | Resource use/economic impact | | | | Life impact | | | |
| --- | --- | --- | --- | --- | --- | --- | --- | --- | --- | --- | --- | --- | --- | --- | --- | --- | --- | --- | --- | --- | --- | --- | --- | --- | --- | --- | --- | --- | --- | --- | --- | --- | --- |
|  | Domain | Diarrhoea | | | | | | | | Clostri-dium difficile  infection | | Gastrointestinal (GI) symptoms | | | | | | | | | | | AE of probiotic | Other clinical  out- comes | | Need for additional medical procedures | | | | Absenteeism | | | Quality of life |
|  | Outcome | Occurrence | Duration | Severity | Infectious diarrhoea | Stool consistency | Frequency of stools | Incubation | Other† | C. difficile diarrhoea | Asymptomatic carriage | Vomiting | Bloating | Nausea | Abdominal pain | Constipation | Taste problems | Lack of appetite | Flatulence | Belching | Symptom scale score | Other ‡ | Adverse events of probiotic | Post-surgery complications | Other§ | Need for i.v. rehydration | Need for antibiotic discontinuation | Need for hospitalization  due to diarrhoea | Other**** | School/day care absence | Hospital stay duration | Missed parental work | Overall health |
| **Study ID** | |  | | | | | | | | | | | | | | | | | | | | | | | | | | | | | | | |
| Ahmad 2013 [1] | | 2 |  |  |  |  |  |  |  |  |  | 2 | 2 |  |  |  |  |  |  |  |  |  |  |  |  |  |  |  |  |  |  |  |  |
| Akcam 2015 [2] | | 2 |  |  |  |  |  |  |  |  |  | 2 |  | 2 | 2 | 2 | 2 | 2 |  | 2 |  |  |  |  |  |  |  |  |  |  |  |  |  |
| Arvola 1999 [3] | | 1 | 2 | 2 | 2 |  |  |  |  | 2 |  |  |  |  |  |  |  |  |  |  |  |  |  |  |  |  |  |  |  |  |  |  |  |
| Bau 2020 [4] | | **3** | 3 |  |  |  |  | 3 |  |  |  |  |  |  | 3 | 3 |  |  |  |  |  |  |  |  |  |  |  |  |  |  |  |  |  |
| Basnet 2017 [5] | | **1** | 2 |  |  |  |  |  |  |  |  |  |  |  |  |  |  |  |  |  |  |  | 2 |  |  | 2 | 2 | 2 |  |  |  |  |  |
| Bin 2015 [6] | | 1 | 2 | 2 |  |  |  | 2 | 2 |  |  |  |  |  |  |  |  |  |  |  |  |  |  |  | 2 |  |  |  |  |  |  |  |  |
| Correa 2005 [7] | | **1** | 2 |  | 2 |  |  | 2 | 2 |  |  |  |  |  |  |  |  |  |  |  |  |  | 2 |  |  |  |  |  |  |  |  |  |  |
| Dharani 2017 [8] | | 2 |  |  |  |  |  |  |  |  |  | 2 |  |  |  |  |  |  | 2 |  |  | 2 | 2 |  |  |  |  |  |  |  |  |  |  |
| Erdeve 2004 [9] | | **1** |  |  |  |  |  |  |  |  |  |  |  |  |  |  |  |  |  |  |  |  |  |  |  |  |  |  |  |  |  |  |  |
| Esposito 2018 [10] | | **1** | 2 |  |  | 2 |  |  |  |  |  |  |  |  |  |  |  |  |  |  |  |  |  | 2 |  |  |  |  | 2 |  | 2 |  |  |
| Fox 2015 [11] | | 1 |  | 1 |  |  |  | 2 | 2 |  |  | 2 |  | 2 | 2 |  |  | 2 |  |  |  |  | 2 |  | 2 |  |  |  |  |  |  |  |  |
| Georgieva 2015 [12] | | 1 |  | 2 |  |  |  |  | 2 |  | 2 | 2 | 2 | 2 | 2 | 2 |  |  | 2 |  |  | 2 |  |  |  |  |  |  |  |  |  |  |  |
| Hurduc 2009 [13] | | 2 |  |  |  |  |  |  |  |  |  |  | 2 | 2 | 2 | 2 | 2 | 2 |  |  |  |  |  |  | 2 |  |  |  |  |  |  |  |  |
| Jindal 2017 [14] | | **1** |  |  |  |  |  |  |  |  |  |  |  |  |  |  |  |  |  |  |  |  | 2 |  |  |  |  |  |  |  |  |  |  |
| Jirapinyo 2002 [15] | | 1 | 2 |  |  |  |  | 2 |  |  |  |  |  |  |  |  |  |  |  |  |  |  |  |  |  |  |  |  |  |  |  |  |  |
| Kołodziej 2018 [16] | | **1** |  |  | 2 |  |  |  |  | 2 |  |  |  |  |  |  |  |  |  |  |  |  | 2 |  |  | 2 | 2 | 2 |  |  |  |  |  |
| Korpela 2016 [17] | | 2 |  |  |  |  |  |  |  |  |  |  | 2 |  | 2 | 2 |  |  | 2 |  |  |  |  |  |  |  |  |  |  |  |  |  |  |
| Kotowska 2005 [18] | | **1** |  |  | 2 |  |  |  |  | 2 |  |  |  |  |  |  |  |  |  |  |  |  | 2 |  |  | 2 | 2 | 2 |  |  |  |  |  |
| Lionetti 2006 [19] | |  |  |  |  |  |  |  |  |  |  | 2 | 2 | 2 | 2 | 2 | 2 | 2 | 2 |  | 1 |  | 2 |  |  |  |  |  |  |  |  |  |  |
| Merenstein 2009 [20] | | **1** |  |  |  | 2 |  |  |  |  |  | 2 |  |  | 2 | 2 |  |  |  |  |  |  | 2 |  | 2 |  |  |  |  | 2 |  | 2 | 2 |
| Okazaki 2016 [21] | |  |  |  |  |  |  |  |  |  |  |  |  |  |  |  |  |  |  |  |  | 1 | 2 | 2 |  |  |  |  |  |  |  |  |  |
| Olek 2017 [22] | | 1 & **2** |  |  |  | 2 |  |  |  |  |  | 2 |  |  | 2 |  |  |  | 2 |  |  |  | 2 |  |  |  |  |  |  |  |  |  |  |
| Plewińska 2006 [23] | | 2 |  |  |  |  |  |  |  |  |  | 2 |  | 2 | 2 |  | 2 |  |  |  |  |  |  |  |  |  |  |  |  |  |  |  |  |
| Ranasinghe 2008 [24] | | 1 |  |  |  |  |  |  |  |  |  |  |  |  |  |  |  |  |  |  |  |  |  |  |  |  |  |  |  |  |  |  |  |
| Ruszczyński 2008 [25] | | **1** |  |  | 2 |  |  |  |  | 2 |  |  |  |  |  |  |  |  |  |  |  |  | 2 |  |  | 2 | 2 | 2 |  |  |  |  |  |
| Seki 2003 [26] | | 2 |  |  |  |  |  |  |  |  |  |  |  |  |  |  |  |  |  |  |  |  |  |  |  |  |  |  |  |  |  |  |  |
| Shahraki 2017 [27] | |  |  |  |  |  |  |  |  |  |  | 2 |  |  | 2 |  |  |  | 2 |  |  |  |  |  |  |  |  |  |  |  |  |  |  |
| Shan 2013 [28] | | 1 & **2** | 1 |  |  |  |  |  |  | 2 |  |  |  |  |  |  |  |  |  |  |  |  | 2 |  |  |  |  |  |  |  |  |  |  |
| Sykora 2005 [29] | | 2 |  |  |  |  |  |  |  |  |  | 2 |  | 2 | 2 |  |  |  |  |  |  |  | 2 |  | 2 |  |  |  |  |  |  |  |  |
| Szajewska 2009 [30] | | 2 |  |  |  |  |  |  |  |  |  | 2 |  | 2 | 2 | 2 | 2 | 2 | 2 |  |  |  | 2 |  |  |  | 2 |  |  |  |  |  |  |
| Szymański 2008 [31] | | 1 |  |  |  |  | 2 |  |  |  |  |  |  |  |  |  |  |  |  |  |  |  | 2 |  |  | 2 | 2 | 2 |  |  |  |  |  |
| Tankanow 1990 [32] | | 1 |  |  |  |  |  |  |  |  |  | 2 | 2 |  |  | 2 |  |  |  | 2 |  |  | 2 |  |  |  |  |  |  |  |  |  |  |
| Tolone 2012 [33] | | 2 |  |  |  |  |  |  |  |  |  | 2 |  | 2 | 2 | 2 |  |  |  |  |  |  |  |  |  |  |  |  |  |  |  |  |  |
| Vanderhoof 1999 [34] | | 2 | 2 |  |  | 2 | 2 |  |  |  |  | 2 | 2 | 2 | 2 |  |  | 2 |  |  |  |  |  |  |  |  |  |  |  |  |  |  |  |
| Wang 2014 [35] | | 2 |  |  |  | 2 |  |  |  |  |  | 2 |  | 2 | 2 |  |  | 2 |  |  |  |  |  |  |  |  |  |  |  |  |  |  |  |
| Zakordonets 2016 [36] | | **1** |  |  |  |  |  |  |  |  |  |  |  |  |  |  |  |  |  |  |  |  | 2 |  |  |  |  |  |  |  |  |  |  |
| Zoppi 2001 [37] | |  |  |  |  |  | 2 |  |  |  |  |  |  |  |  |  |  |  |  |  |  | 2 |  |  |  |  |  |  |  |  |  |  |  |

**Table legend:** 1 = primary outcome; 2 = secondary outcome or undefined; 3 = outcome in an observational study; numbers in bold – outcomes declared by the authors as “antibiotic-associated diarrhoea”.

†Including: efficacy of diarrhoea treatment (Bin 2015), diarrhoea-associated dehydration (Correa 2005), time to first occurrence of loose stool (Fox 2015), and mild diarrhoea (Georgieva 2015).

 ‡Including: “abdominal discomfort” (Dharani 2017), symptoms from Gastrointestinal Symptom Rating Scale (Georgieva 2015), “gastrointestinal complications” (Okazaki 2016), “intestinal complaints” (Zoppi 2001).

§Including: compliance with antibiotic treatment (Bin 2015), headache (Fox 2015, Hurduc 2009, Sykora 2005), fatigue (Hurduc 2009), runny nose, cough, earaches, fever, irritability, and lethargy (Merenstein 2009).

¶Including: duration of hospital stay, number of needed postoperative wound dressings (Esposito 2018).

AE = adverse events

1. Ahmad K, Fatemeh F, Mehri N, Maryam S. Probiotics for the treatment of pediatric helicobacter pylori infection: a randomized double blind clinical trial. Iranian journal of pediatrics. 2013;23(1):79-84.

2. Akcam M, Koca T, Salman H, Karahan N. The effects of probiotics on treatment of Helicobacter pylori eradication in children. Saudi medical journal. 2015;36(3):286-90. doi: <https://dx.doi.org/10.15537/smj.2015.3.10124>.

3. Arvola T, Laiho K, Torkkeli S, Mykkanen H, Salminen S, Maunula L, et al. Prophylactic Lactobacillus GG reduces antibiotic-associated diarrhea in children with respiratory infections: a randomized study. Pediatrics. 1999;104(5):e64.

4. Baù M, Moretti A, Bertoni E, Vazzoler V, Luini C, Agosti M. Risk and Protective Factors for Gastrointestinal Symptoms associated with Antibiotic Treatment in Children: A Population Study. Pediatric Gastroenterology, Hepatology & Nutrition. 2020;23:35. doi: 10.5223/pghn.2020.23.1.35.

5. Basnet S, Gauchan E, Adhikari S, Sathian B. Probiotics in the prevention of antibiotic associated diarrhoea in a tertiary teaching hospital in pokhara: A prospective study. Journal of Clinical and Diagnostic Research. 2017;11(10):SC11-SC3. doi: 10.7860/JCDR/2017/25936.10777.

6. Bin Z, Ya-Zheng X, Zhao-Hui D, Bo C, Li-Rong J, Vandenplas Y. The Efficacy of Saccharomyces boulardii CNCM I-745 in Addition to Standard Helicobacter pylori Eradication Treatment in Children. Pediatric gastroenterology, hepatology & nutrition. 2015;18(1):17-22. doi: <https://dx.doi.org/10.5223/pghn.2015.18.1.17>.

7. Corrêa NB, Péret Filho LA, Penna FJ, Lima FM, Nicoli JR. A randomized formula controlled trial of Bifidobacterium lactis and Streptococcus thermophilus for prevention of antibiotic-associated diarrhea in infants. Journal of clinical gastroenterology. 2005;39(5):385‐9. PubMed PMID: CN-00521370.

8. Dharani Sudha G, Nirmala P, Ramanathan R, Samuel V. Comparative study of efficacy and safety of azithromycin alone and in combination with probiotic in the treatment of impetigo in children. International Journal of Current Pharmaceutical Research. 2017;9(6):52-5. doi: 10.22159/ijcpr.2017v9i6.23429.

9. Erdeve O, Tiras U, Dallar Y. The probiotic effect of Saccharomyces boulardii in a pediatric age group. Journal of tropical pediatrics. 2004;50(4):234-6. doi: <https://dx.doi.org/10.1093/tropej/50.4.234>.

10. Esposito C, Roberti A, Turra F, Cerulo M, Severino G, Settimi A, et al. Frequency of Antibiotic-Associated Diarrhea and Related Complications in Pediatric Patients Who Underwent Hypospadias Repair: a Comparative Study Using Probiotics vs Placebo. Probiotics and antimicrobial proteins. 2018;10(2):323-8. doi: <https://dx.doi.org/10.1007/s12602-017-9324-4>.

11. Fox MJ, Ahuja KD, Robertson IK, Ball MJ, Eri RD. Can probiotic yogurt prevent diarrhoea in children on antibiotics? A double-blind, randomised, placebo-controlled study. BMJ open. 2015;5(1):e006474. doi: 10.1136/bmjopen-2014-006474. PubMed PMID: CN-01111087.

12. Georgieva M, Pancheva R, Rasheva N, Usheva N, Ivanova L, Koleva K. Use of the probiotic Lactobacillus reuteri DSM 17938 in the prevention of antibioticassociated infections in hospitalized bulgarian children: a randomized, controlled trial. Journal of IMAB - annual proceeding (scientific papers). 2015;21(4):895‐900. doi: 10.5272/jimab.2015214.895. PubMed PMID: CN-01133218.

13. Hurduc V, Plesca D, Dragomir D, Sajin M, Vandenplas Y. A randomized, open trial evaluating the effect of Saccharomyces boulardii on the eradication rate of Helicobacter pylori infection in children. Acta paediatrica (Oslo, Norway : 1992). 2009;98(1):127-31. doi: <https://dx.doi.org/10.1111/j.1651-2227.2008.00977.x>.

14. Jindal M, Goyal Y, Lata S, Sharma RK. Preventive role of probiotic in antibiotic associated diarrhoea in children. Indian Journal of Public Health Research and Development. 2017;8(3):66-9. doi: 10.5958/0976-5506.2017.00162.0.

15. Jirapinyo P, Densupsoontorn N, Thamonsiri N, Wongarn R. Prevention of antibiotic-associated diarrhea in infants by probiotics. Journal of the Medical Association of Thailand = Chotmaihet thangphaet. 2002;85 Suppl 2:S739-42.

16. Kolodziej M, Szajewska H. Lactobacillus reuteri DSM 17938 in the prevention of antibiotic-associated diarrhoea in children: a randomized clinical trial. Clinical microbiology and infection : the official publication of the European Society of Clinical Microbiology and Infectious Diseases. 2018. doi: <https://dx.doi.org/10.1016/j.cmi.2018.08.017>.

17. Korpela K, Salonen A, Virta LJ, Kumpu M, Kekkonen RA, de Vos WM. Lactobacillus rhamnosus GG Intake Modifies Preschool Children's Intestinal Microbiota, Alleviates Penicillin-Associated Changes, and Reduces Antibiotic Use. PloS one. 2016;11(4):e0154012. doi: <https://dx.doi.org/10.1371/journal.pone.0154012>.

18. Kotowska M, Albrecht P, Szajewska H. Saccharomyces boulardii in the prevention of antibiotic-associated diarrhoea in children: a randomized double-blind placebo-controlled trial. Alimentary pharmacology & therapeutics. 2005;21(5):583-90.

19. Lionetti E, Miniello VL, Castellaneta SP, Magista AM, de Canio A, Maurogiovanni G, et al. Lactobacillus reuteri therapy to reduce side-effects during anti-Helicobacter pylori treatment in children: a randomized placebo controlled trial. Alimentary pharmacology & therapeutics. 2006;24(10):1461-8.

20. Merenstein DJ, Foster J, D'Amico F. A randomized clinical trial measuring the influence of kefir on antibiotic-associated diarrhea: the measuring the influence of Kefir (MILK) Study. Archives of pediatrics & adolescent medicine. 2009;163(8):750-4. doi: <https://dx.doi.org/10.1001/archpediatrics.2009.119>.

21. Okazaki T, Asahara T, Yamataka A, Ogasawara Y, Lane GJ, Nomoto K, et al. Intestinal Microbiota in Pediatric Surgical Cases Administered Bifidobacterium Breve: a Randomized Controlled Trial. Journal of pediatric gastroenterology and nutrition. 2016;63(1):46‐50. doi: 10.1097/mpg.0000000000001140. PubMed PMID: CN-01165832.

22. Olek A, Woynarowski M, Ahren IL, Kierkus J, Socha P, Larsson N, et al. Efficacy and Safety of Lactobacillus plantarum DSM 9843 (LP299V) in the Prevention of Antibiotic-Associated Gastrointestinal Symptoms in Children-Randomized, Double-Blind, Placebo-Controlled Study. The Journal of pediatrics. 2017;186:82-6. doi: <https://dx.doi.org/10.1016/j.jpeds.2017.03.047>.

23. Plewinska EM, Planeta-Malecka I, Bak-Romaniszyn L, Czkwianlanc E, Malecka-Panas E. Probiotics in the treatment of Helicobacter pylori infection in children. Gastroenterologia polska. 2006;13(4):315‐9. PubMed PMID: CN-00623178.

24. Ranasinghe J, Gamlath G, Samitha S, Abeygunawardena A. Prophylactic use of yoghurt reduces antibiotic induced diarrhoea in children. Sri Lanka Journal of Child Health. 2008;36(2):53-6. doi: <http://doi.org/10.4038/sljch.v36i2.50>.

25. Ruszczynski M, Radzikowski A, Szajewska H. Clinical trial: effectiveness of Lactobacillus rhamnosus (strains E/N, Oxy and Pen) in the prevention of antibiotic-associated diarrhoea in children. Alimentary pharmacology & therapeutics. 2008;28(1):154-61. doi: <https://dx.doi.org/10.1111/j.1365-2036.2008.03714.x>.

26. Seki H, Shiohara M, Matsumura T, Miyagawa N, Tanaka M, Komiyama A, et al. Prevention of antibiotic-associated diarrhea in children by Clostridium butyricum MIYAIRI. Pediatr Int. 2003;45(1):86-90. Epub 2003/03/26. PubMed PMID: 12654076.

27. Shahraki T, Shahraki M, Shahri ES, Mohammadi M. No significant impact of Lactobacillus reuteri on eradication of Helicobacter pylori in children (double-blind randomized clinical trial). Iranian red crescent medical journal. 2017;19(3) (no pagination). doi: 10.5812/ircmj.42101. PubMed PMID: CN-01366602.

28. Shan LS, Hou P, Wang ZJ, Liu FR, Chen N, Shu LH, et al. Prevention and treatment of diarrhoea with Saccharomyces boulardii in children with acute lower respiratory tract infections. Beneficial microbes. 2013;4(4):329‐34. doi: 10.3920/bm2013.0008. PubMed PMID: CN-00959577.

29. Sykora J, Valeckova K, Amlerova J, Siala K, Dedek P, Watkins S, et al. Effects of a specially designed fermented milk product containing probiotic Lactobacillus casei DN-114 001 and the eradication of H. pylori in children: a prospective randomized double-blind study. Journal of clinical gastroenterology. 2005;39(8):692-8.

30. Szajewska H, Albrecht P, Topczewska-Cabanek A. Randomized, double-blind, placebo-controlled trial: effect of lactobacillus GG supplementation on Helicobacter pylori eradication rates and side effects during treatment in children. Journal of pediatric gastroenterology and nutrition. 2009;48(4):431-6.

31. Szymanski H, Armanska M, Kowalska-Duplaga K, Szajewska H. Bifidobacterium longum PL03, Lactobacillus rhamnosus KL53A, and Lactobacillus plantarum PL02 in the prevention of antibiotic-associated diarrhea in children: a randomized controlled pilot trial. Digestion. 2008;78(1):13-7. doi: <https://dx.doi.org/10.1159/000151300>.

32. Tankanow RM, Ross MB, Ertel IJ, Dickinson DG, McCormick LS, Garfinkel JF. A double-blind, placebo-controlled study of the efficacy of Lactinex in the prophylaxis of amoxicillin-induced diarrhea. DICP : the annals of pharmacotherapy. 1990;24(4):382-4.

33. Tolone S, Pellino V, Vitaliti G, Lanzafame A, Tolone C. Evaluation of Helicobacter Pylori eradication in pediatric patients by triple therapy plus lactoferrin and probiotics compared to triple therapy alone. Italian journal of pediatrics. 2012;38:63. doi: <https://dx.doi.org/10.1186/1824-7288-38-63>.

34. Vanderhoof JA, Whitney DB, Antonson DL, Hanner TL, Lupo JV, Young RJ. Lactobacillus GG in the prevention of antibiotic-associated diarrhea in children. The Journal of pediatrics. 1999;135(5):564-8.

35. Wang YH, Huang Y. Effect of Lactobacillus acidophilus and Bifidobacterium bifidum supplementation to standard triple therapy on Helicobacter pylori eradication and dynamic changes in intestinal flora. World journal of microbiology & biotechnology. 2014;30(3):847‐53. doi: 10.1007/s11274-013-1490-2. PubMed PMID: CN-01014256.

36. Zakordonets L, Tolstanova G, Yankovskiy D, Dyment H, Kramarev S. Different regimes of multiprobiotic for prevention of immediate and delayed side effects of antibiotic therapy in children. Research journal of pharmaceutical, biological and chemical sciences. 2016;7(3):2194‐201. PubMed PMID: CN-01167212.

37. Zoppi G, Cinquetti M, Benini A, Bonamini E, Bertazzoni E. Modulation of the intestinal ecosystem by probiotics and lactulose in children during treatment with ceftriaxone. Current Therapeutic Research-clinical and Experimental - CURR THER RES. 2001;62:418-35. doi: 10.1016/S0011-393X(01)89006-8.
