# Supplementary material for: Probiotics for the prevention of antibiotic-associated adverse events in children—A scoping review to inform development of a core outcome set
Source: PLoS One. 2020 May 29;15(5):e0228824. doi: 10.1371/journal.pone.0228824 (PMC7259577; doi:10.1371/journal.pone.0228824)
Supplement: S5 Table — (DOCX) [file pone.0228824.s006.docx]

**S5 Table.** Characteristics of the identified outcomes.

| Study ID | Main diarrhea - related outcome measure | Diarrhea definition | Measurement instrument | Outcome assessment timeline | Other outcome measures |
| --- | --- | --- | --- | --- | --- |
| Ahmad 2013 [1] | Diarrhea | Not provided. | No measurement instruments used, diarrhea was reported by patient or parent during routine phone calls. | Outcome assessed once in a week, during the treatment (one week of antibiotic therapy + 3 weeks of omeprazole) and follow-up. Duration of follow-up unclear. | Other GI symptoms patient- or parent-reported, not defined. |
| Akcam 2015 [2] | Diarrhea | Not provided. | No measurement instruments used, patient-reported outcome during routine check-ups. | Outcome assessed on days 0, 7, 14, and 28 of the study (during antibiotic treatment + 2 week follow-up). | Other GI symptoms patient- or parent-reported, not defined. |
| Arvola 1999 [3] | Diarrhea | At least three watery or loose stools per day for a minimum of 2 consecutive days. | Symptom diary with three levels of stool consistency (solid, loose, watery). | First 2 weeks after the beginning of the antimicrobial treatment. | Definition of diarrhea severity: comparison of the stool frequency and stool consistency (solid, loose, watery) between groups.  Diarrhea duration not defined.  In case of diarrhea, microbiological tests were performed: immune assay for adenovirus, rotavirus and C. difficile toxin A, fecal cultures for Salmonella, Shigella, Yersinia, Campylobacter, Clostridium difficile, Staphylococcus aureus, and yeasts,  PCR for Norwalk-like (genogroup I and II) caliciviruses and astroviruses. AAD diagnosis not dependent on the results of those tests. |
| Basnet 2017 [4] | AAD | At least three loose/liquid stools per day. | Parents asked to record the stool frequency and consistency. No specific instrument used. | 5 days after initiation of therapy. | Diarrhea duration not defined. Adverse events: Any other side effects or complaints the parents might have observed during the course of treatment. |
| Bau 2020 [5] | AAD | Presence of 3 or more loose or liquid bowel movements per day during antibiotic treatment or within 14 days from the antibiotics course excluding other etiologies. | No specific stool form assessment tool used.  Some participants were tested for rota- and norovirus infection. | During antibiotic treatment and for additional 14 days. | Antibiotic-associated abdominal pain: presence of abdominal pain not reduced by defecation and not related to other recognizable conditions/fussiness and persisting crying without any other obvious causes in infants and younger children.  Antibiotic-associated constipation: presence of at least 2 of the following: diﬃcult or painful evacuation,  hard or voluminous stools, and need to use a laxative or enema.  Duration and onset of: diarrhea, abdominal pain and constipation not defined. |
| Bin 2015 [6] | Diarrhea | An increase in the frequency of bowel movements (>3/day) or decrease in stool consistency (BSFS score 5 or 6). | BSFS | During antibiotic treatment. | Diarrhea onset: time between the inclusion  of the patient and the onset of diarrhoea  Diarrhea duration: number of days until normalization of stool consistency (BSF score ＜4) and frequency (＜3 stools/day).  Efficacy of diarrhea treatment: 3 categories – “significantly effective” (diarrhea cessation within 72 hours of treatment, and systemic symptoms disappear); “effective” (appearance and frequency of the stool markedly improve within 72 hours of treatment, and systemic symptoms markedly improve), and “ineffective” (appearance and frequency of the stool and systemic symptoms do not improve, or even worse within 72 hours of treatment).  Diarrhea severity: based on occurrence of differently severe types of diarrhea – “diarrhea without dehydration or toxic symptoms”; “diarrhea with moderate to severe dehydration, or with obvious toxic symptoms and signs”. |
| Correa 2005 [7] | AAD | A change in bowel habits with the passage of three or more liquid stools per day for at least 2 consecutive days. | Tools and other definitions not described. | Stools recorded daily for 30 days. | Fecal samples from all patients with diarrhea and one among 3 patients without diarrhea were tested for rotavirus and enteric strains of adenovirus by enzyme immunoassay. AAD diagnosis not dependent on the results of those tests.  Definitions of duration, incubation and diarrhea-associated dehydration were not provided. |
| Dharani 2017  [8] | Diarrhea | Not provided | No measurement instruments used, patient-reported outcome during routine check-up. | During antibiotic treatment. | Abdominal discomfort, flatulence, vomiting |
| Erdeve 2004 [9] | AAD | Watery stools more than twice a day. | Patients were re-evaluated and were questioned about watery stools three or more times on any day of the treatment. | Duration of antibiotic treatment. |  |
| Esposito 2018 [10] | AAD | 3 or more liquid stools (BSFS type 7) in 24 h. | BSFS | Evaluated the patients on each day of hospitalization. | Diarrhea duration: “number of continuous days of diarrhea”.  Consistency: comparison between groups regarding number of stools of BSFS type: <3, 3-5 and >5 respectively.  Other outcomes: duration of hospital stay, postoperative complications, number of needed postoperative wound dressings. |
| Fox 2015 [11] | Diarrhea | Various deﬁnitions of diarrhea. (A) stool consistency≥5 and frequency≥2/day for more than 2 days; (B) stool consistency≥5 and frequency≥3/day for more than 2 days; (C) stool consistency≥6 and stool frequency≥2/day for more than 2 days; and (D) stool consistency≥6 and stool frequency≥3/day for more than 2 days. | Study diary. BSFS. | Duration of antibiotic treatment + 1 week. | Diarrhea incubation – various definitions: 1) time to occurrence of >=2 stools per day; 2 ) time to occurrence of >=3 stools per day; 3) time to first occurrence of stool consistency >=6; 4) time to first occurrence of stool consistency >=5  Diarrhea severity assessment based on comparison between different types of diarrhea (definitions provided in the column “definitions of diarrhea”). |
| Georgieva 2015 [12] | Diarrhea | Three or more soft and not formed or watery bowel movements per day for at least 48 hours. | BSFS. Study diary. | During and up to 21 days post antibiotic treatment. | Mild diarrhea: Any soft and not formed or watery bowel movements.  Diarrhea severity: total number of soft and not formed or watery bowel movements during an episode of diarrhea and the presence of blood and mucus in feces.  Frequency of stool samples positive for C. difficile toxin A and B.  Frequencies of other gastrointestinal symptoms during the study period according to GSRS. |
| Hurduc 2009 [13] | Diarrhea | Self-reported diarrhea, not defined beyond that. | No measurement instruments described. | Assessment on day 28 after the start of the treatment. | Other GI symptoms (abdominal pain, constipation, bloating, taste disturbance, nausea) reported, but not defined. No measurement instruments used. |
| Jindal 2017 [14] | AAD | Self-reported diarrhea, not defined beyond that. | “The frequency and consistency of stool was enquired and noted at each visit”, no measurement instruments described. | Until 14 day after the start of antibiotic therapy. |  |
| Jirapinyo 2002 [15] | Diarrhea | Diarrhea not defined. | “Characteristics and frequency of stools were recorded”. | Not described. | Incubation and duration of diarrhea – not defined. |
| Kołodziej 2018 [16] | AAD & diarrhea | Various definitions: 1) three or more loose or watery stools per day for a minimum of 48 h  2) three or more loose or watery stools per day for a minimum of 24h  3) two or more loose or watery stools per day for a minimum of 24 h.  AAD was diagnosed in cases of diarrhea, defined clinically as above, caused by C. difficile or for otherwise unexplained origin (i.e., negative laboratory stool tests for infectious agents). | BSFS. Amsterdam infant stool scale. Rapid, qualitative, chromatographic  immunoassay that simultaneously detects rotaviruses, adenoviruses  and noroviruses. Stool culture to identify bacterial pathogens (Salmonella spp., Shigella spp., Campylobacter spp., Yersinia spp.). Clostridium difﬁcile toxins A and B were identiﬁed by standard enzyme immunoassay. | Throughout antibiotic treatment. | Additionally: need for i.v. rehydration, need for antibiotic discontinuation, need for hospitalization to manage diarrhea. |
| Korpela 2016 [17] | Diarrhea | Not defined. | Daily symptom diaries. | 7 months in total. | Various gastrointestinal and respiratory complaints were marked down in diaries. The frequency of gastrointestinal complaints (pain, bloating, diarrhea, constipation, flatulence) was documented and reported. |
| Kotowska 2005 [18] | AAD & diarrhea | >=3 loose or watery stools per day for a minimum of 48 h. AAD was diagnosed in cases of diarrhea, deﬁned clinically as above, caused by C. difﬁcile or for otherwise unexplained diarrhea. | Study diaries.  The presence of  rotavirus-antigen was investigated in all diarrheal  stool samples using a commercial latex agglutination  test with a rotavirus-speciﬁc monoclonal antibody. Standard  stool cultures were used to screen for bacteria (Salmonella, Shigella), and C. difﬁcile toxins A and B were identiﬁed by enzyme immunoassay. | During and up to two weeks after antibiotic therapy. | Need for i.v. rehydration, need for antibiotic discontinuation, need for hospitalization to manage diarrhea. |
| Lionetti 2006 [19] | Not assessed |  |  | During therapy and for 10 additional days (20 days in total) | GI symptoms according to GSRS – both total score and individual symptoms. |
| Merenstein 2009 [20] | AAD | No definition, parent-reported. | Reported by parents. Study diary. | 5, 10 and 15th day of the study. | Vomiting, stomach pain, constipation, loose stools, runny nose, cough, earaches, fever, irritability, lethargy.  Absences from day care or school owing to illness.  Missed parental work owing to the child being ill.  Overall health: Likert scale.  Adverse events: adverse events were defined either by the parent or healthcare provider as any event that could possibly be related to the study drink. Serious adverse events were defined as any incidence of death, a life-threatening event, hospitalization, prolonged hospital stay, or an event resulting in permanent disability. |
| Okazaki 2016 [21] | Not assessed |  |  | Until 3 weeks after surgery. | Gastrointestinal complications – not defined.  Postoperative infections – not defined. |
| Olek 2017 [22] | AAD | >= 3 loose/watery stools/24 h | BSFS | Up to one week after probiotic/placebo cessation. | Incidence of loose/watery stools and mean number of loose/watery stools according to BSFS.  Pain, vomiting, ﬂatulence, distension – no measurement tools reported. |
| Plewińska 2006 [23] | Diarrhea | Not defined. | Not described. | Not clear, presumably during antibiotic therapy and for 20 next days. | Abdominal pain, taste disturbances, nausea, vomiting – not defined. |
| Ranasinghe 2008 [24] | Diarrhea | a change from the patient's normal bowel habit, with two or more loose or watery stools for at least two days. | Not described | 3 first days of antibiotic therapy |  |
| Ruszczyński 2008 [25] | AAD & diarrhea | >=3 loose or watery stools per day for a minimum of 48 h. AAD: Diarrhea defined as above, caused by C. difﬁcile or otherwise unexplained diarrhea. | Study diary.  Immunoassay that simultaneously detects rotaviruses and adenoviruses  Stool culture for Salmonella spp., Shigella spp., Escherichia coli, Campylobacter spp.  Clostridium difﬁcile toxins A and B were identiﬁed by  enzyme immunoassay. | Until 2 weeks after the end of the antibiotic therapy. | Additionally: need for i.v. rehydration, need for antibiotic discontinuation, need for hospitalization to manage diarrhea. |
| Seki 2003 [26] | Diarrhea | Muddy or watery stool at over threefold the normal daily frequency. | Not described. | Until sixth day of antibiotic therapy. |  |
| Shahraki 2017 [27] | Not assessed |  |  | Day 0 and 4 weeks after completion of the treatment. | Vomiting, abdominal pain, flatulence and halitosis: questionnaire with symptom rating scale (0 = no symptom, 1 = mild, 2=moderate, 3=severe). |
| Shan 2013 [28] | AAD & diarrhea | >=3 loose or watery stools (5,6 or 7 on the BSFS) during 2 consecutive days caused by C. difficile or of unknown aetiology. | BSFS  Immunoassay for rotavirus antigen and C. difficile toxin A and B. Cultures for Salmonella, Shigella, Campylobacter, Yersinia, Escherichia. | Up to 2 weeks after the end of antibiotic therapy. | Diarrhea duration: From first diarrheic stool to first normal (BSFS score 4 or less) stool. |
| Sykora 2005 [29] | Diarrhea | Not defined. | Reported at follow-up visit, no instruments specified. | A follow-up visit after the treatment period and 4 weeks after  stopping treatment. | Nausea, abdominal pain, vomiting, headache – not defined. |
| Szajewska 2009 [30] | Treatment-associated diarrhea | 3 or more loose or watery stools per day for a minimum of 48 hours occurring during and/or up to 2 weeks after the end of the therapy. | Study diary. Microbiological tests to exclude infectious origin – specific tests not described. | Up to 2 weeks after the end of the therapy. | Abdominal pain, nausea, vomiting, constipation, flatulence, taste disturbance, or loss of appetite – patient-reported, not defined, noted in study diary.  Need for antibiotic therapy discontinuation. |
| Szymański 2008 [31] | Diarrhea | 3 or more loose or watery stools per day for a minimum of 48 h. | Study diary. | during and/or up to 2 weeks after the end of the antibiotic therapy. | Number of stools per day.  Need for i.v. rehydration, need for antibiotic discontinuation, need for hospitalization to manage diarrhea. |
| Tankanow 1990 [32] | Diarrhea | Abnormal frequency and liquidity of fecal discharges. One or more abnormally loose bowel movements throughout study period. | Parent-reported on routine telephone contact. | Diarrhea occurrence through days 1 to 10. Days 2-3 and 10-12 day of study – telephone contact. |  |
| Tolone 2012 [33] | Diarrhea | Not defined. | Parent-reported, study diary. | During treatment period. | Constipation, epigastric pain, nausea, vomiting – not defined, reported in study diary. |
| Vanderhoof 1999 [34] | Diarrhea | 2 liquid stools per day on at least 2 observation periods during the course of this study. | The Stool Consistency Continuum. Investigation of diarrhoea causes was to be pursued if clinical presentation suggested an infectious cause (vomiting, abdominal cramping, and loose, bloody frequent stools). | Parents were contacted every 3 days until antibiotic completion or cessation of diarrhoea | Pain intensity: Intensity score based on a visual analogue scale.  Diarrhea duration: not defined.  Occurrence of loose stools: occurrence of stools, which scored < 4 on the consistency continuum.  Stool frequency: determined by counting the number of stools passed during a 24-hour period.  Visible blood in the stool.  Abdominal pain: according to intensity score.  Nausea, vomiting, bloating, appetite suppression: parent-reported. |
| Wang 2014 [35] | Diarrhea | Not defined. | Not specified. | Up to 6 weeks after treatment. | Deformed excrement, nausea, vomiting, abdominal pain and loss of appetite – not defined. |
| Zakordonets 2016 [36] | AAD | At least 3 soft or liquid stools for at least 2 consecutive days. | Microscopic examination of faecal smears, pathogenic microflora examination. Pathogenic microflora examination not described in detail. Stool form measurement instrument not described. | Following 4 weeks after AB cessation. |  |
| Zoppi 2001 [37] | Not assessed |  |  | Up to 7 days after discharge. | Bowel movement frequency, intestinal complaints – measurement instruments not specified. |

Table legend: AAD – Antibiotic-associated diarrhea, BSFS –Bristol stool form scale, GSRS – gastrointestinal symptom rating score

References

1. Ahmad K, Fatemeh F, Mehri N, Maryam S. Probiotics for the treatment of pediatric helicobacter pylori infection: a randomized double blind clinical trial. Iranian journal of pediatrics. 2013;23(1):79-84.

2. Akcam M, Koca T, Salman H, Karahan N. The effects of probiotics on treatment of Helicobacter pylori eradication in children. Saudi medical journal. 2015;36(3):286-90. doi: <https://dx.doi.org/10.15537/smj.2015.3.10124>.

3. Arvola T, Laiho K, Torkkeli S, Mykkanen H, Salminen S, Maunula L, et al. Prophylactic Lactobacillus GG reduces antibiotic-associated diarrhea in children with respiratory infections: a randomized study. Pediatrics. 1999;104(5):e64.

4. Basnet S, Gauchan E, Adhikari S, Sathian B. Probiotics in the prevention of antibiotic associated diarrhoea in a tertiary teaching hospital in pokhara: A prospective study. Journal of Clinical and Diagnostic Research. 2017;11(10):SC11-SC3. doi: 10.7860/JCDR/2017/25936.10777.

5. Baù M, Moretti A, Bertoni E, Vazzoler V, Luini C, Agosti M. Risk and Protective Factors for Gastrointestinal Symptoms associated with Antibiotic Treatment in Children: A Population Study. Pediatric Gastroenterology, Hepatology & Nutrition. 2020;23:35. doi: 10.5223/pghn.2020.23.1.35.

6. Bin Z, Ya-Zheng X, Zhao-Hui D, Bo C, Li-Rong J, Vandenplas Y. The Efficacy of Saccharomyces boulardii CNCM I-745 in Addition to Standard Helicobacter pylori Eradication Treatment in Children. Pediatric gastroenterology, hepatology & nutrition. 2015;18(1):17-22. doi: <https://dx.doi.org/10.5223/pghn.2015.18.1.17>.

7. Corrêa NB, Péret Filho LA, Penna FJ, Lima FM, Nicoli JR. A randomized formula controlled trial of Bifidobacterium lactis and Streptococcus thermophilus for prevention of antibiotic-associated diarrhea in infants. Journal of clinical gastroenterology. 2005;39(5):385‐9. PubMed PMID: CN-00521370.

8. Dharani Sudha G, Nirmala P, Ramanathan R, Samuel V. Comparative study of efficacy and safety of azithromycin alone and in combination with probiotic in the treatment of impetigo in children. International Journal of Current Pharmaceutical Research. 2017;9(6):52-5. doi: 10.22159/ijcpr.2017v9i6.23429.

9. Erdeve O, Tiras U, Dallar Y. The probiotic effect of Saccharomyces boulardii in a pediatric age group. Journal of tropical pediatrics. 2004;50(4):234-6. doi: <https://dx.doi.org/10.1093/tropej/50.4.234>.

10. Esposito C, Roberti A, Turra F, Cerulo M, Severino G, Settimi A, et al. Frequency of Antibiotic-Associated Diarrhea and Related Complications in Pediatric Patients Who Underwent Hypospadias Repair: a Comparative Study Using Probiotics vs Placebo. Probiotics and antimicrobial proteins. 2018;10(2):323-8. doi: <https://dx.doi.org/10.1007/s12602-017-9324-4>.

11. Fox MJ, Ahuja KD, Robertson IK, Ball MJ, Eri RD. Can probiotic yogurt prevent diarrhoea in children on antibiotics? A double-blind, randomised, placebo-controlled study. BMJ open. 2015;5(1):e006474. doi: 10.1136/bmjopen-2014-006474. PubMed PMID: CN-01111087.

12. Georgieva M, Pancheva R, Rasheva N, Usheva N, Ivanova L, Koleva K. Use of the probiotic Lactobacillus reuteri DSM 17938 in the prevention of antibioticassociated infections in hospitalized bulgarian children: a randomized, controlled trial. Journal of IMAB - annual proceeding (scientific papers). 2015;21(4):895‐900. doi: 10.5272/jimab.2015214.895. PubMed PMID: CN-01133218.

13. Hurduc V, Plesca D, Dragomir D, Sajin M, Vandenplas Y. A randomized, open trial evaluating the effect of Saccharomyces boulardii on the eradication rate of Helicobacter pylori infection in children. Acta paediatrica (Oslo, Norway : 1992). 2009;98(1):127-31. doi: <https://dx.doi.org/10.1111/j.1651-2227.2008.00977.x>.

14. Jindal M, Goyal Y, Lata S, Sharma RK. Preventive role of probiotic in antibiotic associated diarrhoea in children. Indian Journal of Public Health Research and Development. 2017;8(3):66-9. doi: 10.5958/0976-5506.2017.00162.0.

15. Jirapinyo P, Densupsoontorn N, Thamonsiri N, Wongarn R. Prevention of antibiotic-associated diarrhea in infants by probiotics. Journal of the Medical Association of Thailand = Chotmaihet thangphaet. 2002;85 Suppl 2:S739-42.

16. Kolodziej M, Szajewska H. Lactobacillus reuteri DSM 17938 in the prevention of antibiotic-associated diarrhoea in children: a randomized clinical trial. Clinical microbiology and infection : the official publication of the European Society of Clinical Microbiology and Infectious Diseases. 2018. doi: <https://dx.doi.org/10.1016/j.cmi.2018.08.017>.

17. Korpela K, Salonen A, Virta LJ, Kumpu M, Kekkonen RA, de Vos WM. Lactobacillus rhamnosus GG Intake Modifies Preschool Children's Intestinal Microbiota, Alleviates Penicillin-Associated Changes, and Reduces Antibiotic Use. PloS one. 2016;11(4):e0154012. doi: <https://dx.doi.org/10.1371/journal.pone.0154012>.

18. Kotowska M, Albrecht P, Szajewska H. Saccharomyces boulardii in the prevention of antibiotic-associated diarrhoea in children: a randomized double-blind placebo-controlled trial. Alimentary pharmacology & therapeutics. 2005;21(5):583-90.

19. Lionetti E, Miniello VL, Castellaneta SP, Magista AM, de Canio A, Maurogiovanni G, et al. Lactobacillus reuteri therapy to reduce side-effects during anti-Helicobacter pylori treatment in children: a randomized placebo controlled trial. Alimentary pharmacology & therapeutics. 2006;24(10):1461-8.

20. Merenstein DJ, Foster J, D'Amico F. A randomized clinical trial measuring the influence of kefir on antibiotic-associated diarrhea: the measuring the influence of Kefir (MILK) Study. Archives of pediatrics & adolescent medicine. 2009;163(8):750-4. doi: <https://dx.doi.org/10.1001/archpediatrics.2009.119>.

21. Okazaki T, Asahara T, Yamataka A, Ogasawara Y, Lane GJ, Nomoto K, et al. Intestinal Microbiota in Pediatric Surgical Cases Administered Bifidobacterium Breve: a Randomized Controlled Trial. Journal of pediatric gastroenterology and nutrition. 2016;63(1):46‐50. doi: 10.1097/mpg.0000000000001140. PubMed PMID: CN-01165832.

22. Olek A, Woynarowski M, Ahren IL, Kierkus J, Socha P, Larsson N, et al. Efficacy and Safety of Lactobacillus plantarum DSM 9843 (LP299V) in the Prevention of Antibiotic-Associated Gastrointestinal Symptoms in Children-Randomized, Double-Blind, Placebo-Controlled Study. The Journal of pediatrics. 2017;186:82-6. doi: <https://dx.doi.org/10.1016/j.jpeds.2017.03.047>.

23. Plewinska EM, Planeta-Malecka I, Bak-Romaniszyn L, Czkwianlanc E, Malecka-Panas E. Probiotics in the treatment of Helicobacter pylori infection in children. Gastroenterologia polska. 2006;13(4):315‐9. PubMed PMID: CN-00623178.

24. Ranasinghe J, Gamlath G, Samitha S, Abeygunawardena A. Prophylactic use of yoghurt reduces antibiotic induced diarrhoea in children. Sri Lanka Journal of Child Health. 2008;36(2):53-6. doi: <http://doi.org/10.4038/sljch.v36i2.50>.

25. Ruszczynski M, Radzikowski A, Szajewska H. Clinical trial: effectiveness of Lactobacillus rhamnosus (strains E/N, Oxy and Pen) in the prevention of antibiotic-associated diarrhoea in children. Alimentary pharmacology & therapeutics. 2008;28(1):154-61. doi: <https://dx.doi.org/10.1111/j.1365-2036.2008.03714.x>.

26. Seki H, Shiohara M, Matsumura T, Miyagawa N, Tanaka M, Komiyama A, et al. Prevention of antibiotic-associated diarrhea in children by Clostridium butyricum MIYAIRI. Pediatr Int. 2003;45(1):86-90. Epub 2003/03/26. PubMed PMID: 12654076.

27. Shahraki T, Shahraki M, Shahri ES, Mohammadi M. No significant impact of Lactobacillus reuteri on eradication of Helicobacter pylori in children (double-blind randomized clinical trial). Iranian red crescent medical journal. 2017;19(3) (no pagination). doi: 10.5812/ircmj.42101. PubMed PMID: CN-01366602.

28. Shan LS, Hou P, Wang ZJ, Liu FR, Chen N, Shu LH, et al. Prevention and treatment of diarrhoea with Saccharomyces boulardii in children with acute lower respiratory tract infections. Beneficial microbes. 2013;4(4):329‐34. doi: 10.3920/bm2013.0008. PubMed PMID: CN-00959577.

29. Sykora J, Valeckova K, Amlerova J, Siala K, Dedek P, Watkins S, et al. Effects of a specially designed fermented milk product containing probiotic Lactobacillus casei DN-114 001 and the eradication of H. pylori in children: a prospective randomized double-blind study. Journal of clinical gastroenterology. 2005;39(8):692-8.

30. Szajewska H, Albrecht P, Topczewska-Cabanek A. Randomized, double-blind, placebo-controlled trial: effect of lactobacillus GG supplementation on Helicobacter pylori eradication rates and side effects during treatment in children. Journal of pediatric gastroenterology and nutrition. 2009;48(4):431-6.

31. Szymanski H, Armanska M, Kowalska-Duplaga K, Szajewska H. Bifidobacterium longum PL03, Lactobacillus rhamnosus KL53A, and Lactobacillus plantarum PL02 in the prevention of antibiotic-associated diarrhea in children: a randomized controlled pilot trial. Digestion. 2008;78(1):13-7. doi: <https://dx.doi.org/10.1159/000151300>.

32. Tankanow RM, Ross MB, Ertel IJ, Dickinson DG, McCormick LS, Garfinkel JF. A double-blind, placebo-controlled study of the efficacy of Lactinex in the prophylaxis of amoxicillin-induced diarrhea. DICP : the annals of pharmacotherapy. 1990;24(4):382-4.

33. Tolone S, Pellino V, Vitaliti G, Lanzafame A, Tolone C. Evaluation of Helicobacter Pylori eradication in pediatric patients by triple therapy plus lactoferrin and probiotics compared to triple therapy alone. Italian journal of pediatrics. 2012;38:63. doi: <https://dx.doi.org/10.1186/1824-7288-38-63>.

34. Vanderhoof JA, Whitney DB, Antonson DL, Hanner TL, Lupo JV, Young RJ. Lactobacillus GG in the prevention of antibiotic-associated diarrhea in children. The Journal of pediatrics. 1999;135(5):564-8.

35. Wang YH, Huang Y. Effect of Lactobacillus acidophilus and Bifidobacterium bifidum supplementation to standard triple therapy on Helicobacter pylori eradication and dynamic changes in intestinal flora. World journal of microbiology & biotechnology. 2014;30(3):847‐53. doi: 10.1007/s11274-013-1490-2. PubMed PMID: CN-01014256.

36. Zakordonets L, Tolstanova G, Yankovskiy D, Dyment H, Kramarev S. Different regimes of multiprobiotic for prevention of immediate and delayed side effects of antibiotic therapy in children. Research journal of pharmaceutical, biological and chemical sciences. 2016;7(3):2194‐201. PubMed PMID: CN-01167212.

37. Zoppi G, Cinquetti M, Benini A, Bonamini E, Bertazzoni E. Modulation of the intestinal ecosystem by probiotics and lactulose in children during treatment with ceftriaxone. Current Therapeutic Research-clinical and Experimental - CURR THER RES. 2001;62:418-35. doi: 10.1016/S0011-393X(01)89006-8.
