# Supplementary material for: Probiotics for the prevention of antibiotic-associated adverse events in children—A scoping review to inform development of a core outcome set
Source: PLoS One. 2020 May 29;15(5):e0228824. doi: 10.1371/journal.pone.0228824 (PMC7259577; doi:10.1371/journal.pone.0228824)
Supplement: S6 Table — (DOCX) [file pone.0228824.s007.docx]

**S6 Table.** Risk of bias assessment for the singe included cohort study

| **Quality Assessment Criteria** | **Criterion to be Fulfilled to Award Asterix (*)** | **Bau 2020** |  |
| --- | --- | --- | --- |
|  |  | **Selection (4*maximum)** | |
| **Representativeness of the exposed cohort** | a) truly representative of the average children in the community *  b) somewhat representative of the average children in the community *****  c) selected group of users eg nurses, volunteers  d) no description of the derivation of the cohort | * |  |
| **Selection of the non exposed cohort** | a) drawn from the same community as the exposed cohort *****  b) drawn from a different source  c) no description of the derivation of the non exposed cohort | * |  |
| **Ascertainment of exposure** | a) secure record (eg surgical records) *****  b) structured interview *****  c) written self report  d) no description | - |  |
| **Demonstration that outcome of interest was not present at start of study** | a) yes *****  b) no | * |  |
|  |  | **Comparability (2*maximum)** | |
| **Comparability of cohorts on the basis of the design or analysis** | 1. The study controls for clearly described confounding factors** 2. No control for, or no adequate description of confounding factors, | - |  |
|  |  | **Outcome (3* maximum)** | |
| **Assessment of outcome** | a) independent blind assessment *****  b) record linkage*****  c) self report  d) no description | - |  |
| **Was follow-up long enough for outcomes to occur?** | a) yes *****  b) no | * |  |
| **Adequacy of follow-up of cohorts** | a) complete follow up - all subjects accounted for *****  b) subjects lost to follow up unlikely to introduce bias - small number lost - <20% follow up, or description provided of those lost *****  c) follow up rate < 20% and no description of those lost  d) no statement | * |  |

Adapted from Newcastle-Ottawa scale (Retrieved from <http://www.ohri.ca/programs/clinical_epidemiology/oxford.asp>)
